# *CUBN* as a Novel Locus for End-Stage Renal Disease: Insights from Renal Transplantation

**DOI:** 10.1371/journal.pone.0036512

**Published:** 2012-05-04

**Authors:** Anna Reznichenko, Harold Snieder, Jacob van den Born, Martin H. de Borst, Jeffrey Damman, Marcory C. R. F. van Dijk, Harry van Goor, Bouke G. Hepkema, Jan-Luuk Hillebrands, Henri G. D. Leuvenink, Jan Niesing, Stephan J. L. Bakker, Marc Seelen, Gerjan Navis, on behalf of the REGaTTA (REnal GeneTics TrAnsplantation) Groningen group

**Affiliations:** 1 Division of Nephrology, Department of Internal Medicine, University Medical Center Groningen, University of Groningen, Groningen, The Netherlands; 2 Unit of Genetic Epidemiology & Bioinformatics, Department of Epidemiology, University Medical Center Groningen, University of Groningen, Groningen, The Netherlands; 3 Department of Surgery, University Medical Center Groningen, University of Groningen, Groningen, The Netherlands; 4 Department of Pathology and Medical Biology, University Medical Center Groningen, University of Groningen, Groningen, The Netherlands; 5 Department of Transplant Immunology, University Medical Center Groningen, University of Groningen, Groningen, The Netherlands; Innsbruck Medical University, Austria

## Abstract

Chronic kidney disease (CKD) is a complex disorder. As genome-wide association studies identified cubilin gene *CUBN* as a locus for albuminuria, and urinary protein loss is a risk factor for progressive CKD, we tested the hypothesis that common genetic variants in *CUBN* are associated with end-stage renal disease (ESRD) and proteinuria. First, a total of 1142 patients with ESRD, admitted for renal transplantation, and 1186 donors were genotyped for SNPs rs7918972 and rs1801239 (case-control study). The rs7918972 minor allele frequency (MAF) was higher in ESRD patients comparing to kidney donors, implicating an increased risk for ESRD (OR 1.39, *p* = 0.0004) in native kidneys. Second, after transplantation recipients were followed for 5.8 [3.8–9.2] years (longitudinal study) documenting ESRD in transplanted kidneys – graft failure (GF). During post-transplant follow-up 92 (9.6%) cases of death-censored GF occurred. Donor rs7918972 MAF, representing genotype of the transplanted kidney, was 16.3% in GF vs 10.7% in cases with functioning graft. Consistently, a multivariate Cox regression analysis showed that donor rs7918972 is a predictor of GF, although statistical significance was not reached (HR 1.53, *p* = 0.055). There was no association of recipient rs7918972 with GF. Rs1801239 was not associated with ESRD or GF. In line with an association with the outcome, donor rs7918972 was associated with elevated proteinuria levels cross-sectionally at 1 year after transplantation. Thus, we identified *CUBN* rs7918972 as a novel risk variant for renal function loss in two independent settings: ESRD in native kidneys and GF in transplanted kidneys.

## Introduction

Chronic kidney disease (CKD) is a complex multifactorial disorder with an important genetic component [1]–[3]. A recent genome-wide association study (GWAS) identified the cubilin gene *CUBN* as a locus for albuminuria: a missense single-nucleotide polymorphism (SNP) rs1801239 (Ile2984Val) in this gene was associated with elevated urinary albumine-to-creatinine ratio and microalbuminuria in both the general population and in diabetic patients [4].

As albuminuria is a risk factor for progression of CKD up to end stage renal disease (ESRD) [5], we hypothesized that genetic variation in *CUBN* is associated with development of ESRD. To test this hypothesis we genotyped patients with ESRD, admitted for renal transplantation, with their donors as a control population, for SNPs in the *CUBN* locus and followed the recipients after transplantation documenting clinical parameters and occurrence of graft failure (GF).

Two *CUBN* SNPs were genotyped in our study: the previously published rs1801239 and a tagSNP rs7918972. The latter was selected based on its linkage disequilibrium with 9 other SNPs thus covering more variability in the locus and taking into account that one of the linked polymorphisms is a coding missense variant which might potentially be functional. Another selection criterion was the minor allele frequency (MAF); we targeted a lower part of the common variability range, with MAFs between 10 and 15%.

Within this cohort we performed essentially two independent analyses: 1) ESRD patients admitted for renal transplantation versus kidney donors (extreme case-control study) – to test for association with ESRD in native kidneys; and 2) long-term post-transplant follow-up for GF in the recipients (longitudinal study) – for association with ESRD in the transplanted kidney.

We also tested association of the *CUBN* SNPs with 24-h total urinary protein excretion as an intermediate phenotype.

## Materials and Methods

### Study population

**Figure 1:**
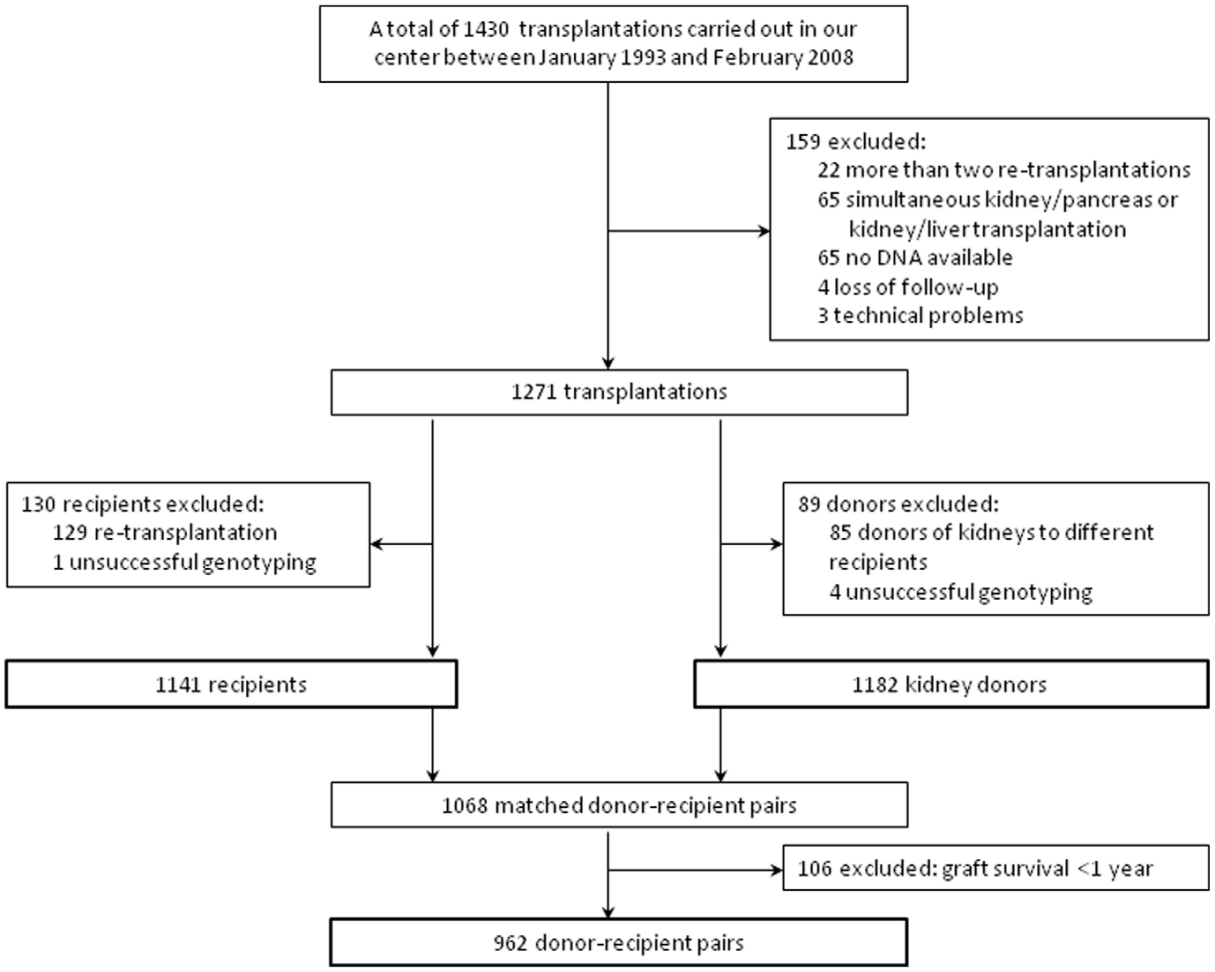
A flowchart of the study participants selection.

From all renal transplantations carried out in our center between 1993 and 2008 we retrospectively selected 1142 first graft recipients and 1186 donors for the present genetic study. The exclusion criteria were: cases of re-transplantation, combined kidney/pancreas or kidney/liver transplantation, technical problems, absence of DNA and loss of follow-up. A flowchart of the study participants selection is shown in the *Figure 1*. After transplantation the recipients were followed up and immunosuppression regimen, clinical and laboratory parameters, and time to GF were documented. GF was defined as return to dialysis or re-transplantation and was censored for death with a functioning graft. Cases with post-transplant graft survival <1 year were excluded from the analyses, to decrease heterogeneity in the sample, as graft loss <1 year is to an important extent due to acute complications, such as technical surgical problems, delayed graft function and/or acute rejection episodes, whereas we wanted to focus on the process of chronic transplant dysfunction. Donor and recipient characteristics, transplantation-related parameters and clinical data (24 h urinary protein excretion, blood pressure, renal function) were retrieved from medical records. The Institutional Review Board of the University Medical Center Groningen approved the study protocol. Written informed consent was given by all recipients and living donors. For deceased donors, with research carried out after the organ removal and implantation, no consent was required. According to Dutch law general consent for organ donation and transplantation includes consent for research projects. The study was conducted according to the principles of the Declaration of Helsinki. All the genetic and clinical data were anonymized prior to analyses.

### DNA isolation, tagSNP selection and genotyping

DNA was extracted from peripheral whole blood (in recipients and living donors) or lymph nodes/spleen lymphocytes (in deceased donors) using a commercial kit following the manufacturer's instructions, transferred into 2 ml Eppendorf tubes and stored at −20°C. Absorbance at 260 nm was measured with NanoDrop spectrophotometer (ND-1000, NanoDrop Technologies) and DNA concentration was calculated by the NanoDrop nucleic acid application module. As a measure of DNA purity 260/280 and 260/230 absorbance ratios were assessed. Where samples failed to meet the minimum DNA concentration and purity recommended for Illumina genotyping, repeated isolation attempts were made.

Two SNPs in the *CUBN* locus were genotyped: missense (Ile2984Val) rs1801239 and rs7918972. The latter is a tagSNP in the *CUBN* intron, which was selected using Genome Variation Server v5.11 (Seattle SNPs Program for Genomic Applications). This program utilizes the LDSelect algorithm [6]. All the SNPs within the *CUBN* gene including 500 bases at the gene flanking regions were submitted to the selection procedure. The following parameter settings were used: HapMap-CEU population (unrelated only, no HapMap 3), monomorphic sites excluded, r^2^ threshold 0.8, minimal genotype coverage for tagSNPs 85%. Further, for our study we considered SNPs with MAFs 10–15%, tagging as many other variants as possible including the missense ones. Rationale for the arbitrary MAF cut-off was based on the general expectation that rarer variants have a slightly higher likelihood to be causal and may confer stronger effects. At the same time, as power to detect such effects depends on sample size, we were constrained by the moderate sample size of our study. That is why we set the cut-off in the range of 10–15%. Using these settings, the SNP rs7918972 was the best tagSNP meeting all our criteria (minimal MAF – 10%, maximal number of the tagged SNPs – 9, tagging a missense variant) and therefore was ultimately chosen for this study. This SNP is in strong linkage disequilibrium with intronic SNPs rs4088454, rs7897625, rs7897716, rs7898076, rs11254232, rs11254238, rs7897442, rs7897705 and missense (Asn3552Lys) rs1801232, all of which map to the *CUBN* locus. The LD structure of the studied *CUBN* SNPs is shown in the *Supplemental Figure S1*.

Genotyping of the selected SNPs was performed using the Illumina VeraCode GoldenGate assay kit (Illumina, San Diego, CA, USA), according to the manufacturer's instructions. Genotype clustering and calling were performed using BeadStudio Software (Illumina). In five individuals genotyping was unsuccessful.

### Statistical analysis

Analyses were performed with PASW Statistics 18.0 (SPSS Inc., Chicago, IL) and PLINK v1.07 (S. Purcell, http://pngu.mgh.harvard.edu/purcell/plink/) [7]. QUANTO v1.2.4 (http://hydra.usc.edu/gxe/) and PASS v11 were used for power estimation. PolyPhen2 [8] was used to predict functional consequences of the missense SNP. The studied *CUBN* SNPs LD structure plot was generated with SNAP v2.2 [9].

As a routine data quality control, alleles frequencies, Hardy-Weinberg equilibrium and case/control differential missingness were tested for. Subsequent statistical analyses were performed on a final sample of 2323 subjects in a case-control design (1141 recipients vs 1182 donors) and 962 renal transplant recipients in a longitudinal design. With two-sided p = 0.05, assuming an additive genetic model and MAF of 10–15%, we had 57% and 99% power to detect an OR of 1.2 and 1.4, respectively, in the ESRD case-control analysis, and 43% and 87% power to detect a HR of 1.5 and 2.0, respectively, in the Cox regression analysis of graft survival.

Genotype-phenotype associations were tested under an additive genetic model and results (regression coefficients and *p*-values) are reported per copy of the minor allele.

In the case-control analysis, the PLINK DFAM algorithm was used to account for donor-recipient relatedness within living-donor transplantation cases. Interaction between the SNPs was tested with the PLINK *–epistasis* function which includes the interaction term and the marginal effects of the SNPs into the interaction model. Subsequently, stratified logistic regression analyses were performed for each of the three groups of minor allele carriers of both SNPs using the group of non-carriers as the reference.

For the longitudinal study we included cases with post-transplant graft survival ≥1 year. The effect of SNPs on graft survival was investigated with Kaplan-Meier and Cox regression analyses including known predictors of GF (donor and recipient age and sex, donor type, cold and warm ischemia times, immunosuppressive therapy).

Association between genotypes and 24 h urinary protein excretion was studied cross-sectionally at 1 year after transplantation assuming stable graft function at this time-point. As proteinuria was considered a left-censored phenotype with 0 values in 24.4% of patients (due to the diagnostic assay detection limit and rounding of routinely reported values), it was analyzed with Tobit regression [10], [11], both univariately and including relevant covariates (age, sex, systolic and diastolic blood pressure).

## Results

Main patients characteristics are presented in *Table 1*.

**Table 1:** Main patients and transplantation-related characteristics.

**ESRD patients, n = 1141**	
Age, years	48.2±13.5
Sex: male, n (%)	662 (58.0)
Primary disease:	
- glomerulopathies, n (%)	292 (25.6)
- kidney cysts, n (%)	188 (16.5)
- tubulo-interstitial lesions, n (%)	135 (11.8)
- diabetes types I and II, n (%)	47 (4.1)
- renal hypoplasia, n (%)	23 (2.0)
- drug-induced nephritis, n (%)	15 (1.3)
- other/uncertain etiology, n (%)	488 (43)
**Kidney donors, n = 1182**	
Age, years	44.5±14.3
Sex: male, n (%)	603 (51.0)
Living donors, n (%)	282 (23.9)
- from which related donors, n (%)	164 (58.2)
**Transplantation, n = 962 renal transplant recipients**	
Cold ischemia time, minutes	1140 [869–1428]
Total warm ischemia time, minutes	40 [34–50]
Follow-up duration, years	5.8 [3.8–9.5]
Measured GFR at 1 year post-transplant, ml/min	54.8±19.2
Total proteinuria at 1 year post-transplant, g/24 h	0.20 [0.05–0.40]
Acute rejection episodes history, n (%)	324 (33.7)
Graft failure, n (%)	92 (9.6)
Death with a functioning graft, n (%)	152 (15.8)

Continuous normally distributed variables are presented as mean±SD, non-normally distributed – as median [IQR].

The overall minor allele frequency was 13.1% for rs7918972 and 12.5% for rs1801239. There was no deviation from Hardy-Weinberg equilibrium in controls (*p* = 0.2908 for rs7918972; *p* = 0.4126 for rs1801239). The missing genotypic data fraction was not different between cases and controls (*p* = 1.000 and *p* = 0.625 for rs7918972 and rs1801239, respectively). There was no linkage disequilibrium between rs7918972 and rs1801239 (r^2^ = 0.002, D′ = 0.059). The missense rs1801232 (Asn3552Lys), tagged by rs7918972, was predicted to be benign by PolyPhen2: score 0.011; sensitivity 0.96; specificity 0.72.

### Case-control study: ESRD patients vs kidney donors

The minor allele frequency (MAF) for rs7918972 was significantly higher in ESRD patients as compared to kidney donors, implicating an increased risk of ESRD: OR [95% CI] 1.39 [1.16–1.65], *p* = 0.0004, in an additive model adjusted for age, sex and case-control relatedness *(* *Table 2* *)*; additional adjustment for diabetes status did not change the results. There was no association between rs7918972 genotype and any of the primary diseases (etiology of ESRD). The MAF for rs7918972 was not different between living and deceased donors and in the latter it was not significantly associated with the cause of death (mortality due to cerebro- or cardiovascular accident vs other reasons). Genotype of rs1801239 was not associated with case/control status or any of the other traits studied.

**Table 2:** *CUBN* SNPs in the case-control study of ESRD patients versus kidney donors.

*CUBN* SNPs		ESRD patients, n = 1141	Kidney donors, n = 1182	OR [95% CI] per copy of the minor allele^a^	*p* value^a^
**rs7918972**	Genotypes, count	21/301/819	12/246/924		
	MAF, %	**15.0%**	**11.4%**	**1.39 [1.16–1.65]**	**0.0004**
rs1801239	Genotypes, count	8/276/857	14/266/902		
	MAF, %	12.8%	12.4%	1.04 [0.87–1.24]	0.6686

OR, odds ratio; CI, confidence interval.

a Logistic regression model adjusted for age and sex, with adjustment for case-control relatedness (DFAM algorithm).

The effects of the two SNPs were not independent as a case-control test for epistasis revealed an interaction between them (*p* = 5×10^−10^). A finer analysis showed that the rs7918972 minor allele requires a copy of the rs1801239 minor allele to express its risk phenotype (OR 3.15 [2.21–4.48], *p* = 1.8×10^−10^), whereas the minor allele of rs1801239 displays protective effect in the absence of rs7918972 minor allele (OR 0.65 [0.52–0.81], *p* = 1.7×10^−4^) *[* *Table 3* *]*.

**Table 3:** Interaction between the SNPs in the *CUBN* locus in the case-control study of ESRD patients versus kidney donors.

*CUBN* SNPs	rs7918972		
	N of the minor allele copies	0	1 or 2
		Reference	OR 0.93 [0.75–1.15]
	0	OR 1.00	*p* = 0.484
**rs1801239**		*n = 1352*	n = 407
		OR 0.65 [0.52–0.81]	OR 3.15 [2.21–4.48]
	1 or 2	*p* = 1.7×10^−4^	*p* = 1.8×10^−10^
		n = 391	n = 173

Logistic regression model adjusted for age and sex. Odds ratios (OR) [95% confidence intervals] for risk of ESRD, *p*-values and patients number (n) are presented in relation to simultaneous presence of both minor alleles in genotype.

### Longitudinal study: post-transplant follow-up

A total of 92 (9.6%) cases of death-censored GF occurred and 151 (15.8%) patients died with a functioning graft during a median [IQR] of 5.8 [3.8–9.5] years of follow-up.

**Figure 2:**
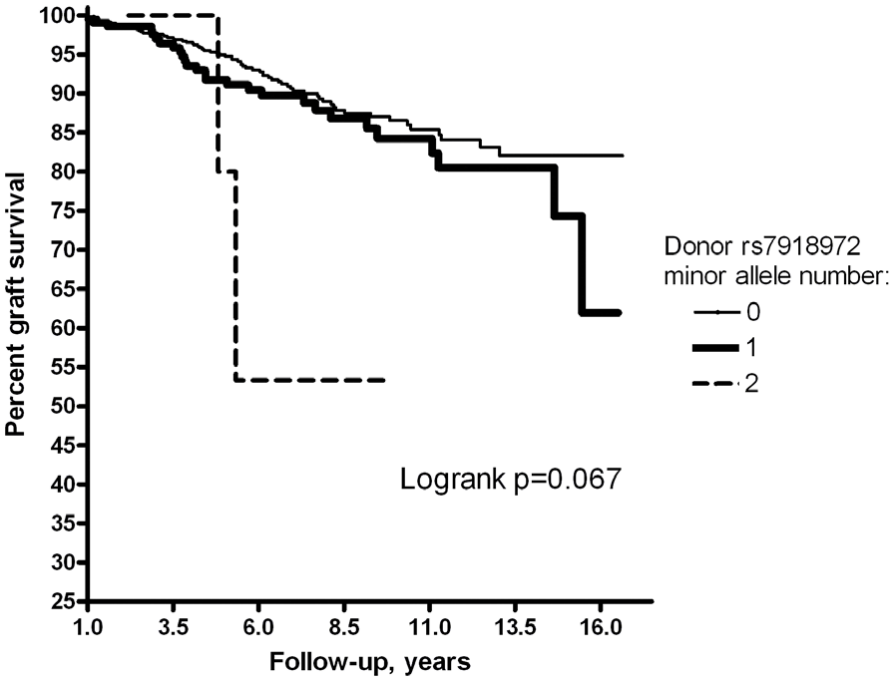
Curves of long-term renal graft survival by donor rs7918972 genotype. Numbers 0 to 2 designate corresponding number of the minor allele copies per genotype. The logrank test showed borderline statistical significance of the differences between the respective curves.

Donor MAF, representing genotype of the transplanted kidney, was higher in subjects that suffered death-censored GF as compared to cases with a functioning graft (16.3% vs 10.7%, respectively). Kaplan-Meier survival analysis revealed worse graft survival (*p* = 0.067) for the carriers of the minor allele *(* *Figure 2* *)*. Consistently, a multivariate Cox regression analysis showed that donor kidney rs7918972 is a predictor of GF yielding a HR of 1.53 [0.99–2.37], *p* = 0.055, per copy of the minor allele, in a model adjusted for donor and recipient age and sex, donor type (living vs deceased), ischemia times, immunosuppressive drug use and acute rejection episodes *(* *Table 4* *)*. In contrast, recipient rs7918972 was not associated with development of GF (HR 1.00, *p* = 0.992). Neither donor nor recipient rs1801239 was significantly associated with GF. There was no statistically significant interaction between the two SNPs in the longitudinal analysis of GF.

**Table 4:** *CUBN* SNPs in the longitudinal study with follow-up for graft failure.

Genotype	*CUBN* SNPs		Graft failure, n = 92	Functioning graft, n = 870	HR [95% CI] per copy of the minor allele^a^	*p* value^a^	HR [95% CI] per copy of the minor allele^b^	*p* value^b^
Donor	**rs7918972**	Genotypes, count	2/26/64	8/171/691				
		MAF, %	**16.3%**	**10.7%**	**1.50 [0.99–2.26]**	**0.056**	**1.53 [0.99–2.37]**	**0.055**
	rs1801239	Genotypes, count	2/15/75	11/201/658				
		MAF, %	10.3%	12.8%	0.80 [0.49–1.30]	0.363	0.75 [0.43–1.31]	0.311
Recipient	rs7918972	Genotypes, count	3/22/67	15/234/621				
		MAF, %	15.2%	15.2%	0.94 [0.62–1.43]	0.773	1.00 [0.64–1.56]	0.992
	rs1801239	Genotypes, count	0/20/72	6/212/651				
		MAF, %	10.9%	12.9%	0.78 [0.48–1.26]	0.308	0.70 [0.40–1.25]	0.229

HR, hazard ratio; CI, confidence interval.

a univariate Cox regression.

b multivariate Cox regression model adjusted for donor and recipient age and sex, donor type (living or deceased), ischemia times, immunosuppressive medication use, and history of acute rejection episodes.

Neither donor nor recipient genotypes were significantly associated with cardiovascular or all-cause mortality during post-transplant follow-up.

We found donor rs7918972 to be associated with proteinuria levels cross-sectionally at 1 year of post-transplant follow-up (beta 0.201, *p* = 0.015) [*Table 5*]. No association between the SNPs and renal function by measured GFR or creatinine clearance was observed at the same time-point; however, donor rs7918972 showed a directionally consistent, although not statistically significant, trend for association with an increased rate of GFR decline (data not shown).

**Table 5:** *CUBN* SNPs association with urinary total protein excretion cross-sectionally at 1 year after transplantation.

Genotype	SNP	Univariate Tobit regression			Multivariate Tobit regression^a^		
		Coefficient	SE	*p* value	Coefficient	SE	*p* value
Donor	**rs7918972**	**0.223**	**0.083**	**0.007**	**0.201**	**0.082**	**0.015**
	rs1801239	−0.039	0.078	0.617	−0.021	0.077	0.784
Recipient	rs7918972	−0.072	0.071	0.313	−0.049	0.070	0.488
	rs1801239	−0.028	0.081	0.726	−0.023	0.080	0.779

a Model adjusted for donor and recipient age and sex, donor type (living or deceased), systolic and diastolic blood pressure. Coefficients are given per copy of the minor allele.

## Discussion

In the present study we followed up the results of a recent GWAS, which identified the cubilin gene *CUBN* as a locus for albuminuria [4]. As albuminuria is an established risk factor for progressive renal function loss, the GWAS findings raised the hypothesis that genetic variation in the *CUBN* locus could be associated with progressive renal function loss and finally end stage renal disease. To test this hypothesis we studied the cited top SNP as well as a tagSNP in *CUBN* in relation to final renal clinical outcomes, namely ESRD in native kidneys and GF in the transplanted kidney.

In a case-control design we studied rs7918972 and rs1801239 genotypes in ESRD patients versus kidney donors. The MAF for rs7918972 was significantly higher in ESRD patients as compared to kidney donors, imposing a 39% increased risk for ESRD per copy of the minor allele. Follow-up data after transplantation showed direction-consistent trend for an association between donor kidney rs7918972 and development of GF in recipients. Thus, the SNP in *CUBN* locus was associated with susceptibility to develop ESRD in two settings, namely ESRD in native kidneys and GF in transplanted kidneys.

Transplantation represents a unique setting, also from genetic point view: an organ with its own genotype functions in an organism with another genotype. We tested both donor and recipient genotype for association with the renal outcome to investigate whether it is the kidney genotype that determines its own fate or it is the recipient genotype that influences function and survival of the transplanted organ. This unique design is useful for genetic research in nephrology as it enables discrimination between the renal and extra-renal mechanisms [12].

In our study, it was donor rather than recipient *CUBN* genotype that was associated with GF, suggesting involvement of local, intra-renal pathways in processes of transplanted kidney survival which are independent of systemic influences.

Albuminuria is known as a predictor of cardiovascular and non-cardiovascular mortality [13]. However, in our study *CUBN* genotypes did not associate with cerebro- or cardiovascular accident as a cause of death in donors and cardiovascular and all-cause mortality after transplantation in recipients.

As no albuminuria data were available and urinary albumin levels are known to correlate with total protein, we tested association of the *CUBN* SNPs with 24-h total urinary protein excretion as a surrogate phenotype. Interestingly, we found donor rs7918972 to be associated with elevated proteinuria levels cross-sectionally at 1 year after transplantation. This is consistent with our results of association with the outcome, and also in line with the results of a recent study which revealed, using exome sequencing, a deleterious mutation in *CUBN* in a family of proteinuric patients, thus confirming the *CUBN* gene involvement in proteinuria [14].

In the original GWAS [4] the *CUBN* SNP rs1801239, associated with elevated urinary albumine-to-creatinine ratio and microalbuminuria. However, this SNP was not associated with CKD or estimated GFR. In agreement with this, our case-control study showed no association between this SNP and ESRD. Also, rs1801239 was not associated with GF in our longitudinal study Instead, it was the other *CUBN* polymorphism, the tagSNP rs7918972, that was associated with ESRD in our study.

The *CUBN* locus is characterized by a high variability, with both common and rare mutations. Mutations in the *CUBN* locus are known to be the cause of Imerslund-Gräsbeck syndrome (OMIM #261100, Finnish type) which is a rare (the estimated prevalence is <6∶1,000,000) autosomal recessive disorder characterized by vitamin B12 deficiency commonly resulting in megaloblastic anemia, and also neurological damage and mild proteinuria [15]. However, we did not aim to address previously clinically-associated Mendelian mutations in the *CUBN* in our study. We aimed to investigate whether *common* variation, as opposed to rare mutations in Imerslund-Gräsbeck syndrome, in the *CUBN* associates with kidney disease. In the same time, we targeted a lower part of the common variability range, with MAFs between 10 and 15%, aiming to reveal allegedly stronger genetic effects. We selected two SNPs in the *CUBN* locus for the present study: first, the one previously published to be associated with albuminuria levels in the general population, i.e. the missense variant (Ile2984Val) rs1801239, and second the tagSNP in the *CUBN* intron, rs7918972. The latter is in high linkage disequilibrium (r^2^ = 0.831) with another missense variant rs1801232 (Asn3552Lys) in *CUBN*, which might be responsible for the biological impact of the polymorphism on the protein level. The minor allele of rs1801232 leads to an asparagine-to-lysine amino acid substitution in the C-terminal CUB27 domain of cubilin. Despite the amino acids differ in chemical properties (isoelectric points: Asn 5.4, Lys 9.8), the substitution was predicted to be benign by bioinformatics algorithms. However, the mutation is close to sites of N-glycosylation (amino acid 3533) and di-sulfide bond (between amino acids 3564 and 3586) and therefore might potentially interfere with secondary protein structure and, consequently, function. The Imerslund-Gräsbeck syndrome mutations, for which functionality was proven, affect the IF-cobalamin-binding region in the CUB8 domain of cubilin (rs121434430 Pro1297Leu), CUB6 domain (*CUBN* IVS6 C-G in-frame insertion) or CUB23 domain (*CUBN* IVS23 G-T transversion at the conserved donor splice site of exon 23). The SNPs that we studied were spatially distant from these variants and located to the CUB22 domain (rs1801239) and CUB27 domain (rs1801232 tagged by rs7918972).

The rs7918972 *CUBN* SNP, associated with ESRD and GF in our study, is localized in high proximity to the neighboring gene, *RSU1*. Although the nine SNPs tagged by rs7918972 are all located in the *CUBN* locus, linkage disequilibrium with and involvement of the *RSU1* is theoretically possible and cannot be entirely ruled out (Suppl. Fig. S1). The *RSU1* gene encodes Ras suppressor protein 1, which participates in the Ras signal transduction pathway, growth inhibition and nerve-growth factor induced differentiation processes. Its mRNA is expressed in the kidney (according to the NCBI GEO profiles), in a low-to-moderate quantity (51 transcripts per million, according to the NCBI EST profiles). However, functional proof is beyond the scope of the present study, and further research will be needed to discriminate between the effects of these neighboring genes.

Interestingly, we found an interaction between the two SNPs studied. According to our data, the rs7918972 minor allele requires a copy of the rs1801239 minor allele to express its risk phenotype, whereas the minor allele of rs1801239 displays protective effect in the absence of rs7918972 minor allele. This pattern was observed in the case-control study and warrants further investigation to determine whether found statistical interaction has biological implications.

Our study was conducted in kidney transplant recipients, and thus reflects a population that developed ESRD in their native kidneys and was eligible to receive a transplantation. As such, renal transplant recipients represent a relatively healthy subset of the ESRD population, a selection bias inherent to any study in renal transplantation. This should be considered a limitation to our study. In chronic renal disease, both in native kidney disease and in transplantation, mortality is high, and for analyses on ESRD the competing risks of mortality, in particular cardiovascular, are therefore relevant to consider. In the current population, mortality with functioning graft was 15.8 percent, and no association with either of the *CUBN* SNPs was observed.

Our longitudinal study of graft failure may have been underpowered to detect a significant SNP effect. Insufficient power might thus be an explanation of the fact that convincing statistical significance was not reached in the graft survival analysis for association with rs7918972. Studies in larger populations are warranted to confirm an association between the *CUBN* SNP and GF.

### Conclusion

Our study confirms association of the *CUBN* with renal phenotypes of progressive renal function loss and urine protein loss. We first identified *CUBN* SNP rs7918972 as a novel genetic variant of susceptibility for ESRD in a case-control design. In a separate proof-of-principle longitudinal study, which served as an internal replication, we reproduced the association. Thus, rs7918972 was associated with susceptibility to develop progressive renal function loss in two settings, namely ESRD in native kidneys and GF in transplanted kidneys. It was kidney genotype that associated with increased risk, supporting impact of intra-renal pathways on organ damage. Our study set-up – analyzing both donor and recipient genotypes – provides a powerful design for hypothesis-driven studies on risk loci for renal damage enabling differentiation between local, intra-renal, and systemic, extra-renal, influences.

## Supporting Information

Figure S1 ***CUBN*** **regional LD plot.** The figure was generated using HapMap data (release 22, CEU population). The horizontal blue line represents an arbitrarily chosen LD threshold (r^2^ = 0.8). SNPs are shown as diamonds. The color gradient between the diamonds reflects the pairwise LD between the SNPs, with color intensity of each diamond being directly proportional to the r^2^ value. Boundaries of the gene coding regions are shown as green horizontal lines. The largest size diamonds represent the present study SNPs. The shaded area designates a span of the gene region tagged by rs7918972.
(DOCX)
