# Supplementary material for: CUBN as a Novel Locus for End-Stage Renal Disease: Insights from Renal Transplantation
Source: PLoS One. 2012 May 4;7(5):e36512. doi: 10.1371/journal.pone.0036512 (PMC3344899; doi:10.1371/journal.pone.0036512)
Supplement: Figure S1 — CUBN regional LD plot. The figure was generated using HapMap data (release 22, CEU population). The horizontal blue line represents an arbitrarily chosen LD threshold (r2 = 0.8). SNPs are shown as diamonds. The color gradient between the diamonds reflects the pairwise LD between the SNPs, with color intensity of each diamond being directly proportional to the r2 value. Boundaries of the gene coding regions are shown as green horizontal lines. The largest size diamonds represent the present study SNPs. The shaded area designates a span of the gene region tagged by rs7918972. (DOCX) [file pone.0036512.s001.docx]

**Supplemental Material**


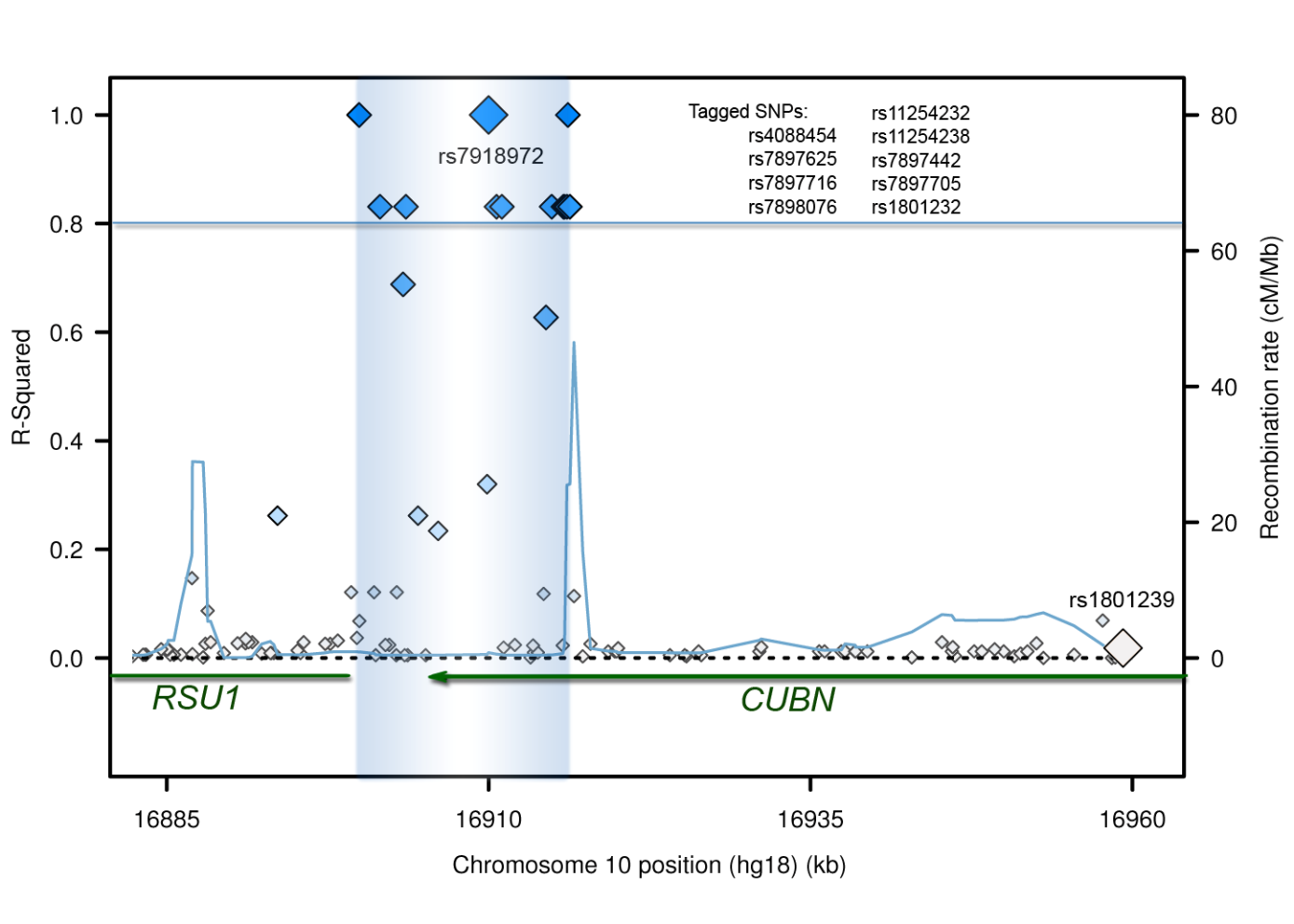


**Figure S1**. ***CUBN* regional LD plot.**

The figure was generated using HapMap data (release 22, CEU population). The horizontal blue line represents an arbitrarily chosen LD threshold (r^2^=0.8). SNPs are shown as diamonds. The color gradient between the diamonds reflects the pairwise LD between the SNPs, with color intensity of each diamond being directly proportional to the r^2^ value. Boundaries of the gene coding regions are shown as green horizontal lines. The largest size diamonds represent the present study SNPs. The shaded area designates a span of the gene region tagged by rs7918972.
